# Reverse Engineering the Neuroblastoma Regulatory Network Uncovers MAX as One of the Master Regulators of Tumor Progression

**DOI:** 10.1371/journal.pone.0082457

**Published:** 2013-12-05

**Authors:** Ricardo D’Oliveira Albanus, Rodrigo Juliani Siqueira Dalmolin, Mauro Antônio Alves Castro, Matheus Augusto de Bittencourt Pasquali, Vitor de Miranda Ramos, Daniel Pens Gelain, José Cláudio Fonseca Moreira

**Affiliations:** Departamento de Bioquímica, Núcleo de Bioinformática, Universidade Federal do Rio Grande do Sul, Porto Alegre, Rio Grande do Sul, Brasil; University of Navarra, Spain

## Abstract

Neuroblastoma is the most common extracranial tumor and a major cause of infant cancer mortality worldwide. Despite its importance, little is known about its molecular mechanisms. A striking feature of this tumor is its clinical heterogeneity. Possible outcomes range from aggressive invasion to other tissues, causing patient death, to spontaneous disease regression or differentiation into benign ganglioneuromas. Several efforts have been made in order to find tumor progression markers. In this work, we have reconstructed the neuroblastoma regulatory network using an information-theoretic approach in order to find genes involved in tumor progression and that could be used as outcome predictors or as therapeutic targets. We have queried the reconstructed neuroblastoma regulatory network using an aggressive neuroblastoma metastasis gene signature in order to find its master regulators (MRs). MRs expression profiles were then investigated in other neuroblastoma datasets so as to detect possible clinical significance. Our analysis pointed MAX as one of the MRs of neuroblastoma progression. We have found that higher MAX expression correlated with favorable patient outcomes. We have also found that MAX expression and protein levels were increased during neuroblastoma SH-SY5Y cells differentiation. We propose that MAX is involved in neuroblastoma progression, possibly increasing cell differentiation by means of regulating the availability of MYC:MAX heterodimers. This mechanism is consistent with the results found in our SH-SY5Y differentiation protocol, suggesting that MAX has a more central role in these cells differentiation than previously reported. Overexpression of MAX has been identified as anti-tumorigenic in other works, but, to our knowledge, this is the first time that the link between the expression of this gene and malignancy was verified under physiological conditions.

## Introduction

Neuroblastoma is the most common extra-cranial solid tumor and one of the leading causes of cancer mortality in children worldwide [1–4]. These tumors are originated from embryonic elements of the neural crest and sympathetic nervous system, usually developing in the adrenal glands, but also arising in nervous tissues of the neck, thorax, abdomen, and pelvis. The metastatic capacity of this type of cancer is notable, being able of compromising almost any tissue in the human body [2,5]. Another distinguishing feature of this disease is its clinical heterogeneity. The possible endpoints span from complete remission to patient death, even with advanced multimodal therapy [6]. Numerous efforts have been made in order to sort neuroblastoma patients in separate risk groups, such as the International Neuroblastoma Risk Group (INRG) staging system [7], which is based on clinical factors and imaging studies, and the Children’s Oncology Group (COG) risk stratification schema, which complements the former with molecular aspects. The molecular approaches for assessing patient clinical outcome are made by detecting DNA copy number alterations or by searching for more specific segmental aberrations, such as chromosomes 11q loss and 17q gain [8,9]. The most reliable molecular classifier, however, is the amplification of the MYCN oncogene, which is linked to grim prognosis in the majority of cases [10–12].

Lower grade neuroblastoma patients can be treated with surgical resection alone, and may even be subject to spontaneous remission without any intervention. Patients with metastatic MYCN amplified tumors have the highest mortality rate and are usually unresponsive to advanced multimodal treatment [6,7,13,14]. Patients with MYCN non-amplified metastatic tumors, however, present highly variable outcomes, but few endpoint predictors have been described for this group. Currently, the best known prognostic indicator is the age at which the tumor is diagnosed [7]. Patients younger than 18 months usually have better prognosis, with more than 90% 6-year event-free survival for patients younger than one year. Patients older than 18 months, on the other hand, suffer higher mortality rates and may present less than 25% 6-years event-free survival [15,16]. Several high-throughput studies have been made over the last years to understand the biology underlying this clinical variability, and at least one of them was aimed exclusively at these metastatic MYCN non-amplified patients [17]. Despite intensive study, few predictors have been brought forward, demanding increased efforts to understand this remarkable disease.

In recent years, the availability of gene expression studies has allowed novel strategies for understanding cancer biology. One of such is the use of mutual information models for inferring the regulatory networks of transcription factors (TFs) and their transcriptional targets (or regulons) in the gene expression profile of a given set of samples [18–20]. This methodology allows the detection of potential causal relationships between TFs and specific cancer signatures. As these statistics depend on rather large sample sizes (*n*>80), only recently are they being successfully applied in biology [21–23]. In this paper, we have reconstructed the neuroblastoma regulatory network in order to find TFs involved in the transition from primary tumors to highly aggressive bone marrow metastasis. We have found evidences that MAX is one of the master regulators of tumor progression, possibly by being an additional regulatory step for the availability of MYC:MAX heterodimers to regulate transcription and increase proliferation. We have also found evidences that MAX plays a more prominent role in SH-SY5Y cells differentiation than previously described.

## Results

### Neuroblastoma regulatory network and master regulator analysis

**Figure 1:**
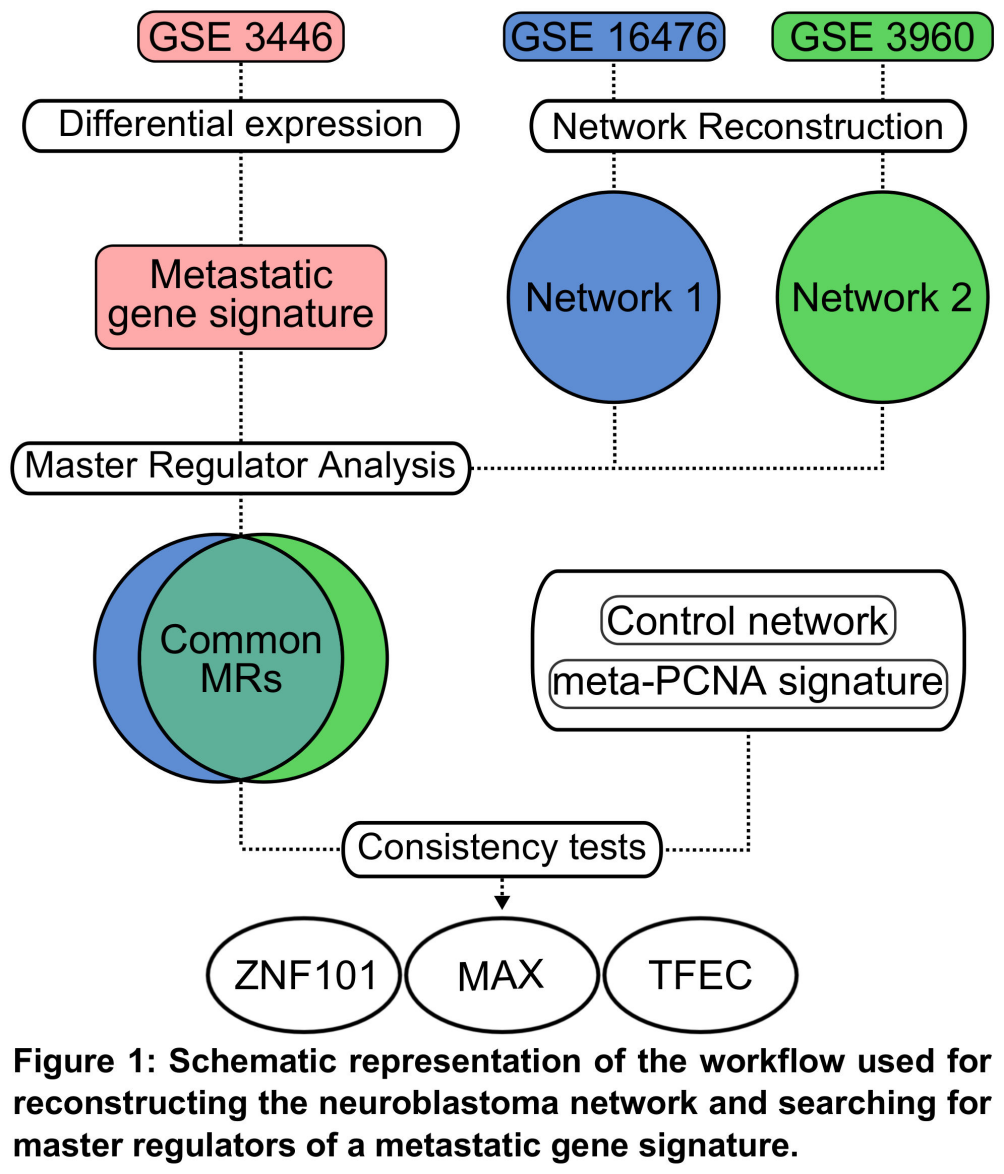
Schematic representation of the workflow used for reconstructing the neuroblastoma network and searching for master regulators of a metastatic gene signature.

**Figure 2:**
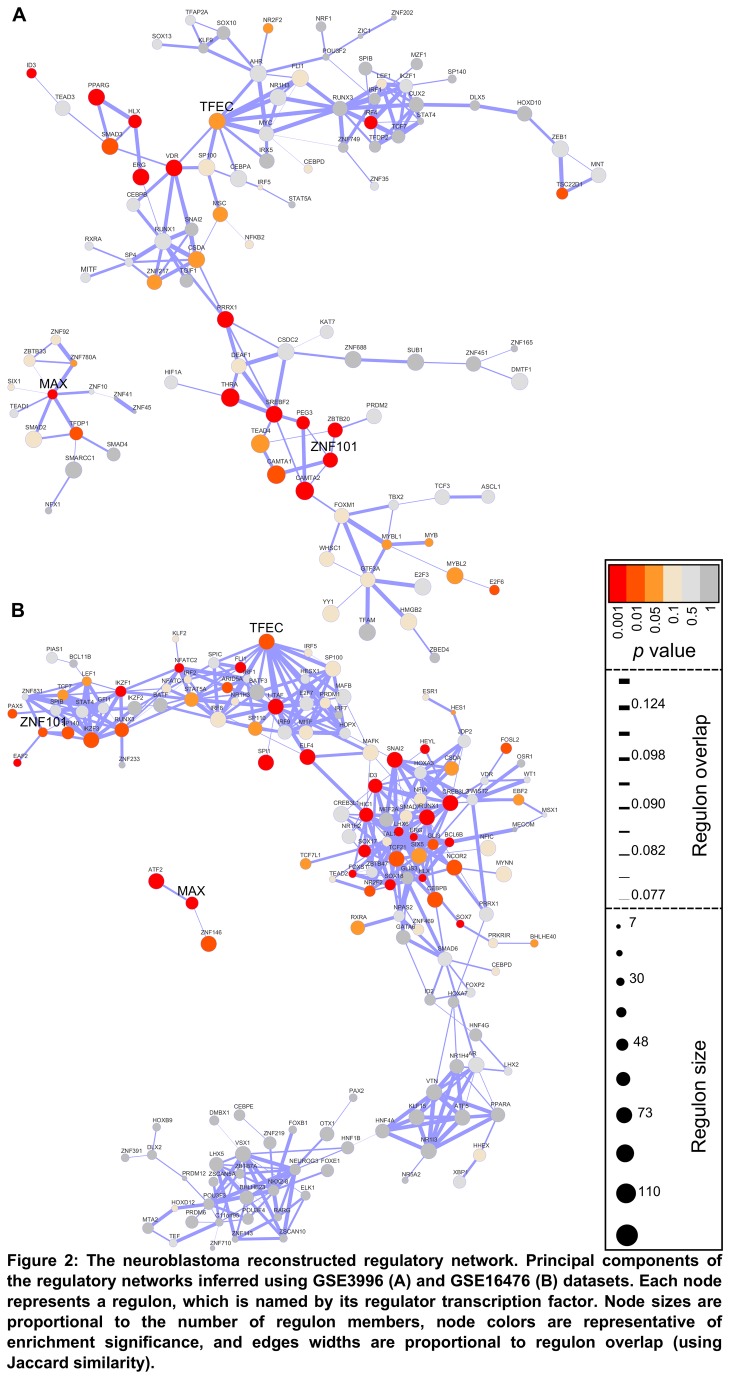
The neuroblastoma reconstructed regulatory network. Principal components of the regulatory networks inferred using GSE3996 (A) and GSE16476 (B) datasets. Each node represents a regulon, which is named by its regulator transcription factor. Node sizes are proportional to the number of regulon members, node colors are representative of enrichment significance, and edges widths are proportional to regulon overlap (using Jaccard similarity).

Through our regulatory network reconstruction workflow (Figure 1), we have identified 15,713 targets for 1,363 TFs in the first reconstructed neuroblastoma regulatory network (GSE16476), and 4,039 targets for 705 TFs in the second (GSE3960) (Figure 2). We have found eight master regulators (MRs) common to both networks (Table S1) using as query an aggressive neuroblastoma metastatic gene signature. We have chosen only regulons common to both networks so as to improve the specificity of our MRs analysis. However, by this criterion alone, there was still an elevated chance of finding nonspecific TFs. To overcome this problem, we have used the metastatic neuroblastoma gene signature to query a healthy mammary tissue network (GSE10780) [24] and use it as a positive control to detect MRs that are not tissue-specific to neuroblastoma. We then re-ran the MR analysis within the two neuroblastoma networks using as query the meta-PCNA proliferative gene signature [25]. This last step was performed in order to exclude MRs that were directly involved with proliferation. Of the eight MRs common to both networks, only MAX, TFEC, and ZNF101 remained after these specificity tests.

### Regulon overlap and motif analysis

In order to validate the power of our pipeline for detecting biologically significant regulons, we have compared the composition of each regulon across the two neuroblastoma networks and verified whether their members were enriched with the binding site motif of its MR. We have found less than 1% overlap in the genes potentially regulated by MAX and ZNF101 in both networks, and 25% for TFEC. We proceeded to search for regulatory motifs in the targets sequences flanking regions. Because there are no available motifs for TFEC and ZNF101 in public databases, we could only query for the known MAX motif V$MAX, obtained at the JASPAR database [26,27]. We have found significant enrichment for V$MAX motif in both networks (*p*=0.038), indicating that our pipeline predicted correctly both MAX regulons, albeit their different compositions. Although there is no public TFEC motif available, this transcription factor is a member of the MITF bHLH family, which is closely related to MAX and MYC families [28]. Members of these families have similar CACGTG binding motifs, meaning that we can query the TFEC regulon using V$MAX motif to determine whether it is regulated by a bHLH transcription factor. TFEC regulon was significantly enriched with this motif (*p*=0.003), suggesting that it is indeed regulated by a bHLH factor.

Given that the MYC/MAX/MAD network may regulate up to 15% of the human genome [29–31], one would not expect finding substantial overlap in both networks, particularly when taking into account the significant genetic heterogeneity across tumors of the same type, which could greatly affect the specificity of such an important regulatory system. Taking this into consideration and the fact that both MAX regulons were significantly enriched with V$MAX binding motif, we chose to carry our analysis with this gene. ZNF101 was not analyzed further because it did not have corresponding probes the datasets below.

### Clinical relevance of MAX and TFEC expression in neuroblastoma

**Figure 3:**
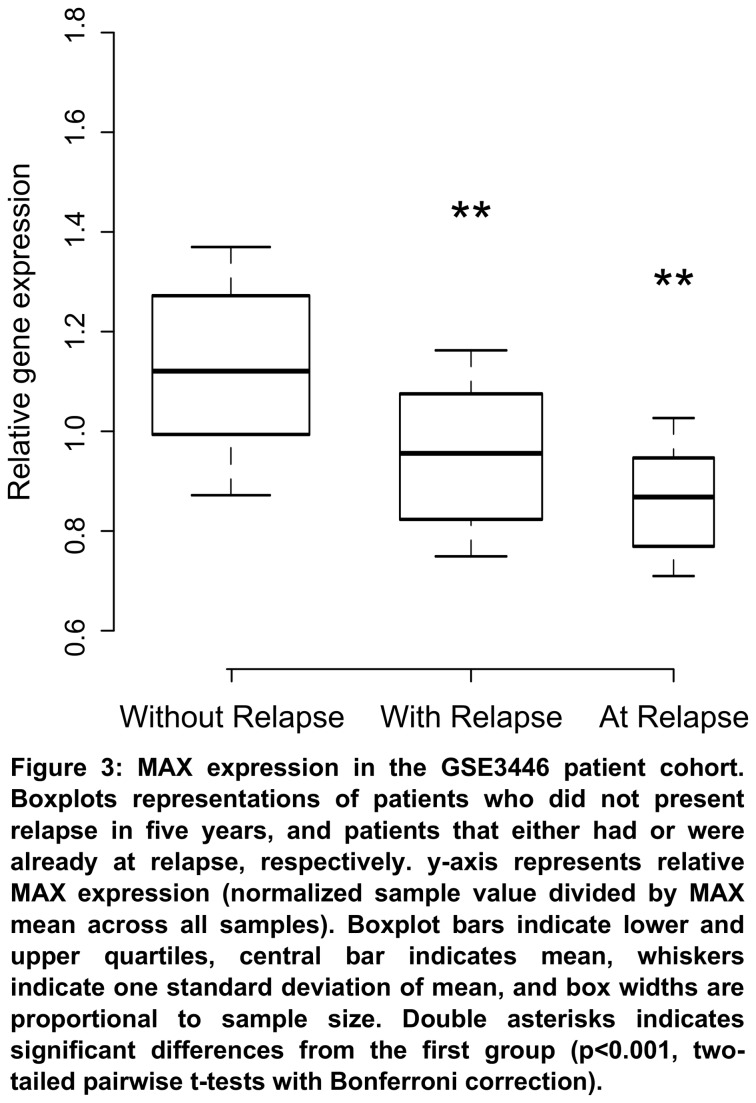
MAX expression in the GSE3446 patient cohort. Boxplots representations of patients who did not present relapse in five years, and patients that either had or were already at relapse, respectively. *y*-axis represents relative MAX expression (normalized sample value divided by MAX mean across all samples). Boxplot bars indicate lower and upper quartiles, central bar indicates mean, whiskers indicate one standard deviation of mean, and box widths are proportional to sample size. Double asterisks indicates significant differences from the first group (*p*<0.001, two-tailed pairwise *t*-tests with Bonferroni correction).

To access whether MAX and TFEC expression could be related to patient outcome, we have analyzed the GSE3446 dataset [17]. This dataset consists of expression profiles of primary tumor biopsies obtained at diagnosis from neuroblastoma patients who (i) either had relapse after five years (*n*=46), (ii) did not present disease progression in the same period (*n*=56), and (iii) from tumors obtained at progression (*n*=12 + 3 obtained both at diagnosis and relapse). All patients in this study had untreated metastatic MYCN non-amplified tumors. 74 of the 102 patients studied at diagnosis were considered high risk by COG risk stratification, and the rest was ranked as intermediate risk. We have found that MAX expression was significantly higher in patients who did not present disease setback in five years as opposed to those who had or were already in relapse (Figure 3), indicating that this gene plays an important role in disease progression. We did not find any significant alterations in TFEC expression.

**Figure 4:**
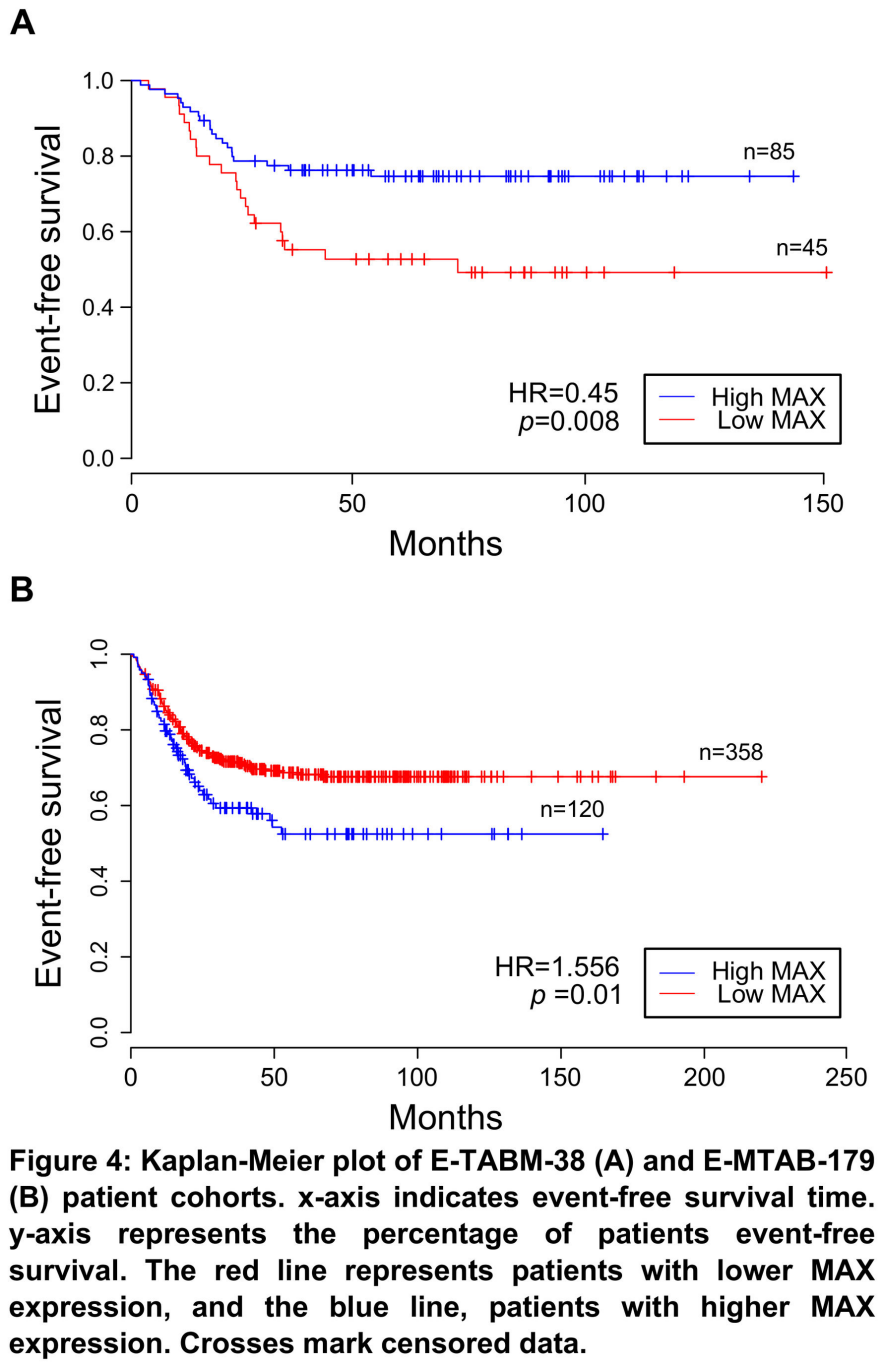
MAX expression and patient survival. Kaplan-Meier plots of E-TABM-38 (A) and E-MTAB-179 (B) patient cohorts. *x*-axis indicates event-free survival time. *y*-axis represents the percentage of patients event-free survival. The red line represents patients with lower MAX expression, and the blue line, patients with higher MAX expression. Crosses mark censored data.

We have also analyzed two neuroblastoma survival cohorts with gene expression data. In the first cohort (E-TABM-38, *n*=130) [32], we have found that lower MAX expression correlated with decreased patient survival (Figure 4a), corroborating our previous result. However, this was not the case with the second cohort (E-MTAB-179, *n*=478) [33], in which higher MAX expression significantly correlated with poor survival (Figure 4b). We could not analyze TFEC because these studies were made with custom array platforms that did not contain probes for this gene. This was also the case with the regulons members themselves, which were poorly represented in these platforms and could not be analyzed further. We chose to analyze these genes in other types of cancers so as to detect if there was a general trend for their expression which could confirm our previous results with MAX and shed some light at TFEC. Using the Kaplan-Meier Plotter web tool [34,35], we have found that higher MAX expression was associated with improved prognosis in breast cancer (Figure S1), in lung cancer (Figure S2), and not related to prognosis in ovarian cancer cohorts (Figure S3). As for TFEC, we have found association with higher expression of this gene and improved lung cancer survival (Figure S4). We have not found associations with TFEC expression in breast and ovarian cancer outcome (Figures S5 and S6, respectively). Detailed results from this analysis are presented in Table 1.

**Table 1:** Survival statistics for MAX and TFEC in other type of cancers.

**Gene**	**Cancer**	**Hazard Ratio**	***p* value**	***n***
MAX	Breast	0.75	1.E-06	2,878
	Lung	0.45	1.E-14	1,404
	Ovarian	ns	ns	1,171
TFEC	Breast	ns	ns	2,878
	Lung	0.57	1.E-09	1,404
	Ovarian	ns	ns	1,171

### Role of MAX and TFEC in SH-SY5Y differentiation

**Figure 5:**
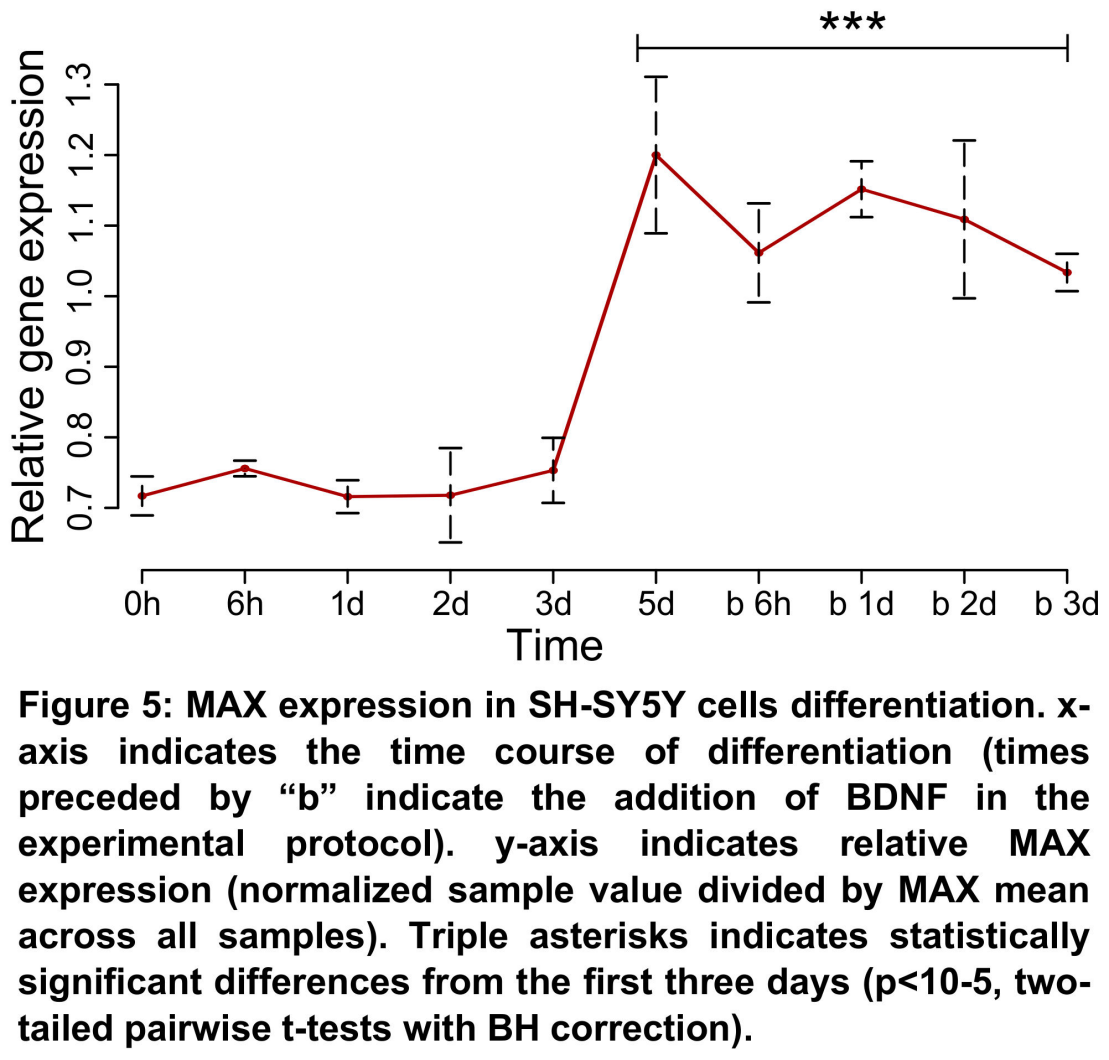
MAX expression in SH-SY5Y cells differentiation. *x*-axis indicates the time course of differentiation (times preceded by “b” indicate the addition of BDNF in the experimental protocol). *y*-axis indicates relative MAX expression (normalized sample value divided by MAX mean across all samples). Triple asterisks indicates statistically significant differences from the first three days (*p*<10^-5^, two-tailed pairwise *t*-tests with BH correction).

To understand whether the alterations detected previously in MAX expression could be involved in neuroblastoma cells differentiation and, as such, provide an explanation to why there seems to be a correlation with patient outcome, we chose to study a dataset of neuroblastoma SH-SY5Y cells undergoing differentiation (GSE9169) [36]. In this study, the authors differentiated cells by treatment with retinoic acid for 8 days, with further addition of brain-derived neurotrophic factor (BDNF) after the 5^th^ day. We have found a significant increase in MAX expression starting at the 3^rd^ and 5^th^ days, which lasted until the end of the differentiation protocol (Figure 5). We have not found any significant alterations in TFEC expression during the course of this experiment (data not shown).

**Figure 6:**
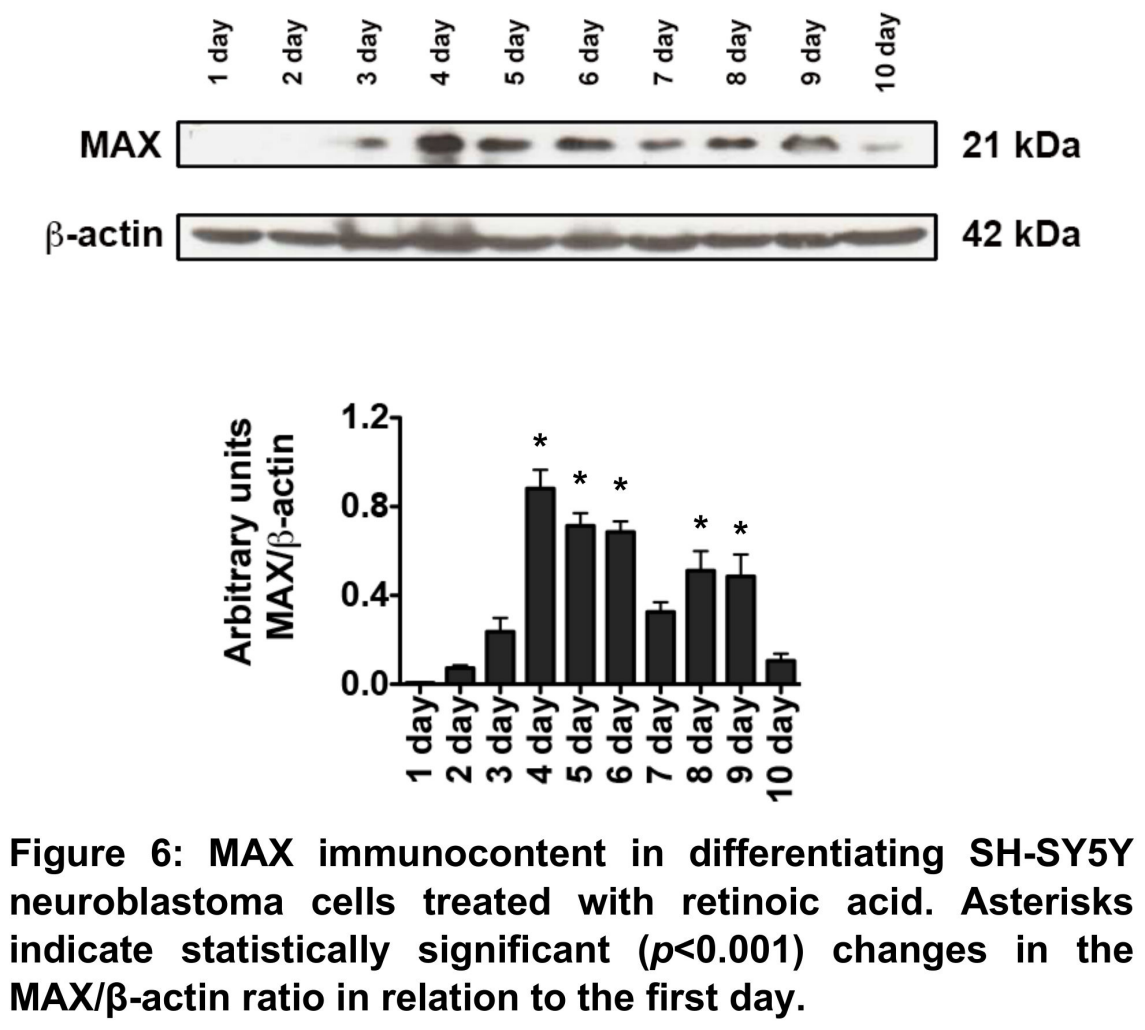
MAX immunocontent in differentiating SH-SY5Y neuroblastoma cells treated with retinoic acid for 10 days. Asterisks indicate statistically significant (*p*<10^-3^) changes in the MAX/β-actin ratio in relation to the first day.

We have also performed a similar SH-SY5Y differentiation protocol in our laboratory and quantified MAX protein content during differentiation. We have found an increased amount of MAX after the 4^th^ day of experiment (Figure 6), demonstrating that the changes in mRNA expression are indeed reflected at protein level in neuroblastoma cells.

## Discussion

Neuroblastoma is clinically very heterogenic, and one of the possible outcomes for this disease is spontaneous tumor regression [1,2]. There are reported cases of differentiation of these tumors into benign ganglioneuromas, but the mechanisms by which this processes can occur are still unknown [8,37,38]. We have queried our reconstructed neuroblastoma network with a genetic signature related to bone marrow metastases, which are considered grim clinical prognosis for neuroblastoma patients [3,39]. We have observed that MAX regulon was significantly enriched with differentially expressed genes, suggesting its role as a master regulator of the transition from primary tumor to metastasis. Also, this regulon was not enriched in a healthy control network, neither involved with a nonspecific proliferative signature, corroborating that it is related to neuroblastoma-specific pathways.

MAX expression was significantly altered in three independent gene expression studies. In the first, we have observed that neuroblastoma patients who did not present disease relapse in five years had significant higher levels of MAX expression than patients who either had or were already at relapse. Of important note is that only 3 of the 15 patients studied at relapse were from those included at the beginning of the study, meaning that we can treat this last group independently. This result suggests that higher MAX levels are associated to improved patient prognosis in patients with high risk, MYCN non-amplified metastatic neuroblastoma. Corroborating this data, we have analyzed a survival cohort of neuroblastoma patients and found that individuals with lower MAX expression had significantly decreased survival rates than the others. However, we have found the opposite results in a second neuroblastoma cohort. In this study, patients with higher MAX expression levels presented lower survival rates than the rest. It is possible that this incongruence is brought by the great variability in the two survival cohorts. Patients in these studies, albeit having mostly MYCN non-amplified tumors, were sorted across all risk stages and were subject to different types of therapy, thus making difficult our attempts at understanding this data. Nevertheless, we have also found positive association of MAX expression and improved prognosis in two independent cohorts of breast and lung cancer patients, corroborating its role in disease progression.

To address whether MAX expression could affect neuroblastoma by activating a differentiation pathway, we have analyzed a dataset of MYCN non-amplified neuroblastoma cells SH-SY5Y subjected to a differentiation protocol. Strikingly, we have found a peak of MAX expression between the third and fifth days of differentiation, lasting to the end of the experiment. This data makes us believe that MAX is one the late effectors SH-SY5Y differentiation, suggesting that its expression is implicated in favorable outcomes for MYCN non-amplified neuroblastoma patients by means of enhancing cell differentiation and/or impairing proliferation. These results are further corroborated by the increase of MAX protein levels we have observed in our SH-SY5Y cells differentiation protocol.

The MYC/MAX/MAD network is a key axis in the cell decision-making for differentiation and proliferation, and it is altered in several types of cancer [40–42]. Much has been said about the oncogenic roles of MYC family genes, but, other than being the obligatory heterodimer of MYC and MAD families of proteins, little is known about how variations in MAX expression can affect cell-cycle progression. In earlier cell differentiation studies, it was found that MAX expression remains constant throughout the process [43] (for a review, see [44] and references therein). One study demonstrates that MAX ^-/-^ mice are subject to early embryonic lethality [45], indicating that this gene plays a vital role in development. These results suggest that this gene is indispensable to differentiation and proliferation processes, albeit only as an accessory part of its network. Lindeman and colleagues [46], however, have elegantly demonstrated that MAX has a more active role decreasing the size and frequency of tumors when overexpressed in lymphoma susceptible mice. In their study, authors have transfected mice lineages with two different MAX transgenes. They observed that both strains presented impaired lymphoproliferation and delayed tumor onset when co-expressed with a highly active MYC transgene. Interestingly, the authors noted that the MAX transgene with higher activity presented more pronounced tumor impairing capacity than the less active one, indicating that MAX has indeed tumor suppressing properties, and that the latter may be dose-dependent. These results are in accordance to related papers that link MAX overexpression with increased differentiation in other cell lines [47,48], and particularly, the work of Peverali and colleagues using neuroblastoma cells [49]. This author demonstrated that retinoic acid-treatment in SK-N-BE neuroblastoma cells overexpressing MAX induced differentiation more than twice as fast as with retinoic acid alone. Curiously, this cells are MYCN amplified, suggesting that MAX expression is sufficient to revert proliferation even in more aggressive neuroblastoma cell variants.

Because these studies were made using overexpression techniques, one may argue that they are not accurate reflections of biological processes occurring at more physiological conditions. The only currently known association with MAX and tumor biology in humans comes from studies of pheochromocytoma and paraganglioma patients. Several independent research centers have observed correlations regarding possible inactivating mutations in MAX exons and the appearance of these tumors [50–54]. These results further suggest the tumor suppressing properties of MAX. To our notice, the spontaneous increase in MAX expression during SH-SY5Y retinoic acid-induced differentiation was the first of this kind to be reported, and leads us to question whether this gene could have a more prominent role in neuroblastoma progression and neuronal cells progenitors development than previously described.

**Figure 7:**
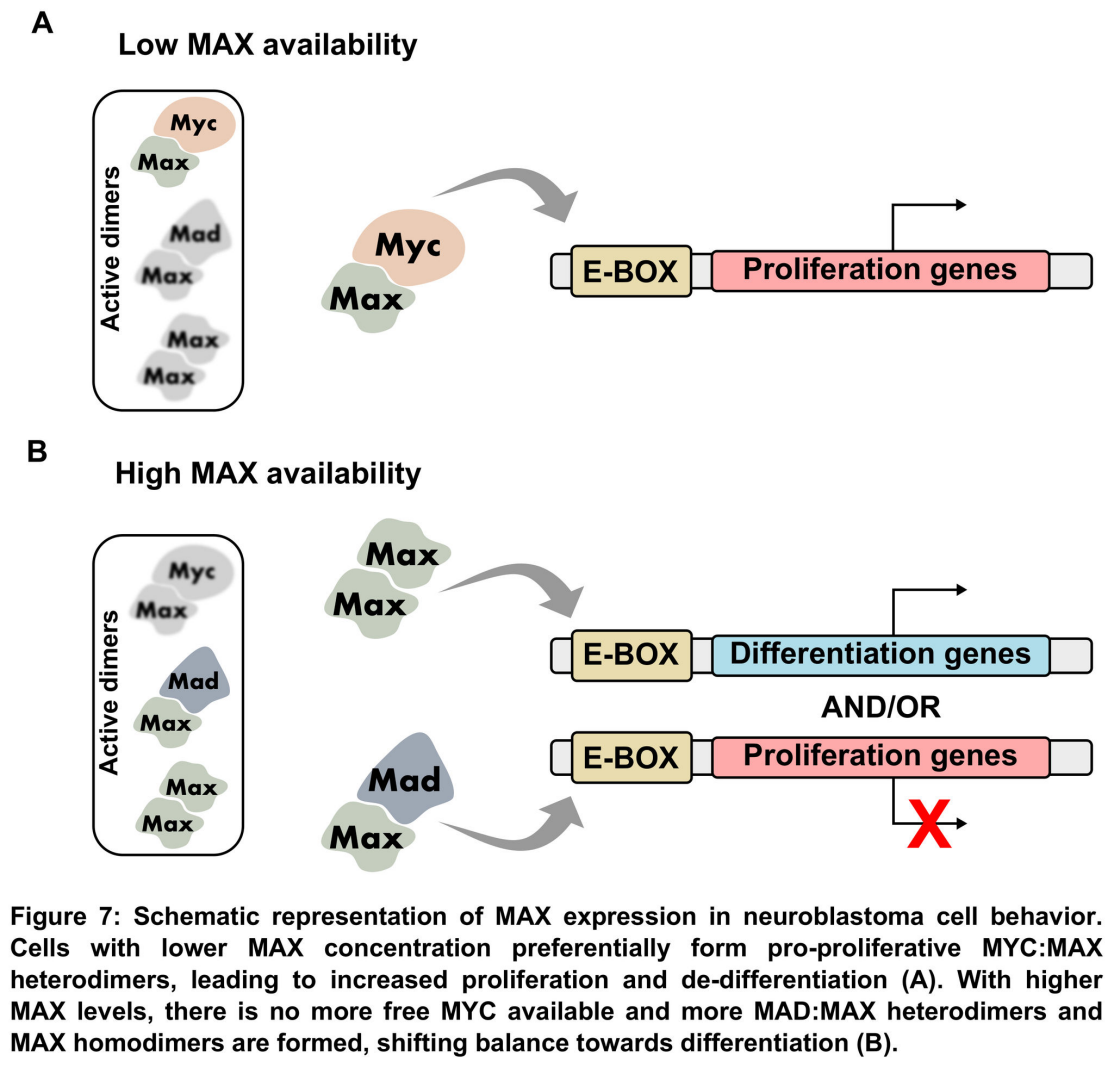
Schematic representation of MAX expression in neuroblastoma cell behavior. Cells with lower MAX concentration preferentially form pro-proliferative MYC:MAX heterodimers, leading to increased proliferation and de-differentiation (A). With higher MAX levels, there is no more free MYC available and more MAD:MAX heterodimers and MAX homodimers are formed, shifting balance towards differentiation (B).

It is known that the MAX:MYC heterodimer induces cell proliferation by interacting with the TRRAP complex and histone acetyltransferases to transcriptionally activate cell cycle activators such as cyclins, and possibly repressing cell cycle inhibitors [55–57]. The MAX:MAD/MNT complex and the MAX homodimer, on the other hand, are proliferation repressors and inducers of terminal differentiation in various cell types [58–60]. The heterodimerization of MYC and MAX is preferential because the stabilization of their bHLH is thermodynamically more favorable than that of MAD and MNT with MAX [61]. Being the limiting factor of its network, one possible mechanism for how changes in MAX expression can interfere directly with proliferation/differentiation fate in cell is by regulating the amount of MYC that can activate gene expression. Lower quantities of MAX would bind preferentially with MYC and change the network balance towards proliferation. As MAX levels rise, all the available MYC binding sites would be occupied and more MAD:MAX, MNT:MAX and MAX:MAX dimers would be formed, switching the balance towards differentiation (Figure 7). In MYCN amplified tumors, a larger fraction of MAX would be used to form pro-proliferative MAX:MYCN heterodimers, explaining how these cells are more resistant to exit division [47]. In line with this, there have been studies with compounds that are able to disrupt the MYC:MAX complex in order to decrease malignancy [62,63]. Of particular relevance is the recent work of Montagne and colleagues [64], that used MAX bHLH as a protein transduction domain for decreasing MYC availability to interact with MAX, thus impairing proliferation in HeLa cells. These results demonstrate MAX capacity of directly interfering with MYC activity. Put together, this data bring MAX forward as a central player in its network, and urge us to dedicate more research in this intriguing subject.

There are questions made during this work that must be addressed in the near future. It would be interesting to compare whether MAX levels are different in stage 4s patients in order to clinically corroborate our findings with SH-SY5Y cells. There is also the putative role of ZNF101 and TFEC, which were pointed as master regulators but could not be implicated in this disease by means of their expression. We have found an indication that the latter is associated in lung cancer outcome, suggesting its importance may be greater than previously thought. The accurate roles of these genes in neuroblastoma progression are an open question and additional assays are needed to address this matter.

## Conclusions

We have found evidences that MAX expression plays a role in neuroblastoma biology that has not been previously described, possibly as an additional regulator of the availability of MYC:MAX heterodimers and the balance of proliferation/differentiation. Our analyses also point that this gene may be used as a candidate predictor for positive clinical outcomes of MYCN non-amplified neuroblastoma patients. We have detected significant associations between higher MAX expression and improved survival rates for breast and lung cancer patients, suggesting that the clinical predictor potential can also be extended to other types of tumors. Lastly, we have observed that MAX expression was significantly altered during SH-SY5Y retinoic acid-induced differentiation, providing a mechanism for which this gene could be linked to tumor progression.

## Materials and Methods

### Neuroblastoma regulatory network reconstruction and master regulator analysis

The neuroblastoma regulatory network was reconstructed and plotted using, respectively, the RTN [65] and RedeR [66] packages for R Statistical Computing, available at Bioconductor [67]. This analysis uses the information-theoretic content (i.e. mutual information) of the gene expression profile for inferring relevant pairwise interactions among genes. Regulatory networks were reconstructed from the neuroblastoma biopsies datasets GSE16476 [68] and GSE3960 [69]. Human transcription factors were gathered from the Animal Transcription Factor Database [70]. Master regulator analyses were made using as query a genetic signature obtained from aggressive metastases. For assessing this metastatic signature, we have used Limma R package [71] on the GSE25623 dataset [72] in order to discover differentially expressed genes between neuroblastoma primary tumors and bone marrow metastases (for a schematic description of this workflow, we once again refer the reader to Figure 1). Master regulators DNA-binding motifs were searched using the FIMO tool for transcription factor binding site prediction [73]. We have considered target genes only those that had at least one motif occurrence in their flanking regions (either 2.5 Kb up and downstream from the gene start codon [74]). To assess whether regulons were significantly enriched with genes regulated by its transcription factor, we verified if our prediction was significantly higher than randomly sampling all human genes 100,000 times using regulon-sized samples. Statistical significance was calculated using one-tailed *z*-tests.

### SH-SY5Y differentiation and Western blot analysis

Human neuroblastoma SH-SY5Y cells were obtained from the American Type Culture Collection (ATCC, Rockville, MD). Cells were cultivated using Dulbecco's Modified Eagle's Medium supplemented with 10% heat-inactivated fetal bovine serum, L-glutamine (2mM), and 0.28 mg/ml of gentamycin sulfate in a 5% CO_2_ humidified incubator at 37°C. Differentiation protocol consisted in reducing fetal bovine serum concentration to 1% and adding retinoic acid (10µM) during 10 days. The culture medium was replaced every three days. Cells were grown in 6-well cluster dishes. 

Proteins (20µg) were separated using SDS-PAGE – 10% (w/v) acrylamide, 0.275% (w/v) bisacrylamide gels – and electrotransferred onto nitrocellulose membranes. Membranes were then incubated in Tris-buffered saline Tween-20 [TBS-T; 20 mM Tris–HCl, pH 7.5, 137 mM NaCl, 0.05% (v/v) Tween 20] for 1h at room temperature. Subsequently, membranes were incubated for 12h with polyclonal rabbit anti-MAX (1:1.000 dilution; Cell Signaling). After washing in TBST, blots were incubated with rabbit peroxidase-linked anti-immunoglobulin G (IgG) antibodies (1:10.000 dilution) for 1.5h at room temperature. Chemiluminescent bands were detected, and densitometric analysis was performed by Image-J® software. All analyses were performed in triplicate.

### Statistical analyses

Unless stated otherwise, all statistical analyses were made using two-tailed *t*-tests. Pairwise *t*-tests were made with Bonferroni and BH *p*-value corrections when more than two groups were analyzed. The latter was used when testing for more than four groups. Survival curves for gene expression were drawn using Kaplan-Meier plot and tested with Cox proportional hazards model using the Survival [75] and Survcomp [76] packages for R statistical computing. The best cutoff for samples split was chosen by plotting all percentiles of gene expression between the upper and lower quartiles and selecting the best performing threshold. For breast, lung and ovarian cancers, statistics were made using the Kaplan-Meier Plotter web tool. Parameters used in all analyses were auto select split cutoff and only the JetSet best probes were selected [77]. All gene expression profiles used in this paper were obtained from Gene Expression Omnibus and ArrayExpress public databases. 

## Supporting Information

Figure S1 **Kaplan-Meier plot of the 2012 breast cancer patient cohort available at the Kaplan-Meier Plotter web tool.** *x*-axis indicates event-free survival time. *y*-axis represents the percentage of patients event-free survival. The black line represents patients with lower MAX expression, and the red line, patients with higher MAX expression. Crosses mark censored data.
(TIFF)

Figure S2 **Kaplan-Meier plot of the unified lung cancer patient cohort available at the Kaplan-Meier Plotter web tool.** *x*-axis indicates event-free survival time. *y*-axis represents the percentage of patients event-free survival. The black line represents patients with lower MAX expression, and the red line, patients with higher MAX expression. Crosses mark censored data.
(TIFF)

Figure S3 **Kaplan-Meier plot of the 2013 ovarian cancer patient cohort available at the Kaplan-Meier Plotter web tool.** *x*-axis indicates event-free survival time. *y*-axis represents the percentage of patients event-free survival. The black line represents patients with lower MAX expression, and the red line, patients with higher MAX expression. Crosses mark censored data.
(TIFF)

Figure S4 **Kaplan-Meier plot of the 2012 breast cancer patient cohort available at the Kaplan-Meier Plotter web tool.** *x*-axis indicates event-free survival time. *y*-axis represents the percentage of patients event-free survival. The black line represents patients with lower TFEC expression, and the red line, patients with higher TFEC expression. Crosses mark censored data.
(TIFF)

Figure S5 **Kaplan-Meier plot of the lung cancer patient cohort available at the Kaplan-Meier Plotter web tool.** *x*-axis indicates event-free survival time. *y*-axis represents the percentage of patients event-free survival. The black line represents patients with lower TFEC expression, and the red line, patients with higher TFEC expression. Crosses mark censored data.
(TIFF)

Figure S6 **Kaplan-Meier plot of the 2013 ovarian cancer patient cohort available at the Kaplan-Meier Plotter web tool.** *x*-axis indicates event-free survival time. *y*-axis represents the percentage of patients event-free survival. The black line represents patients with lower TFEC expression, and the red line, patients with higher TFEC expression. Crosses mark censored data.
(TIFF)

Table S1 **Overview of all Master Regulators found in our study and detailed composition of MAX, TFEC and ZNF101 regulons.**
(XLSX)
