# Supplementary figures and images for: Reverse Engineering the Neuroblastoma Regulatory Network Uncovers MAX as One of the Master Regulators of Tumor Progression
Source: PLoS One. 2013 Dec 5;8(12):e82457. doi: 10.1371/journal.pone.0082457 (PMC3857773; doi:10.1371/journal.pone.0082457)

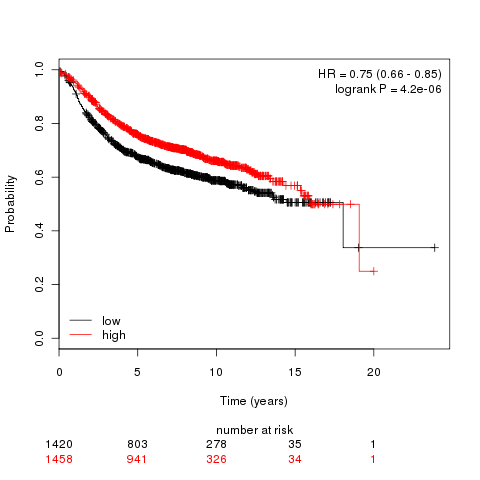

Supplement: Figure S1 — Kaplan-Meier plot of the 2012 breast cancer patient cohort available at the Kaplan-Meier Plotter web tool. x-axis indicates event-free survival time. y-axis represents the percentage of patients event-free survival. The black line represents patients with lower MAX expression, and the red line, patients with higher MAX expression. Crosses mark censored data. (TIFF) [file pone.0082457.s001.tiff]

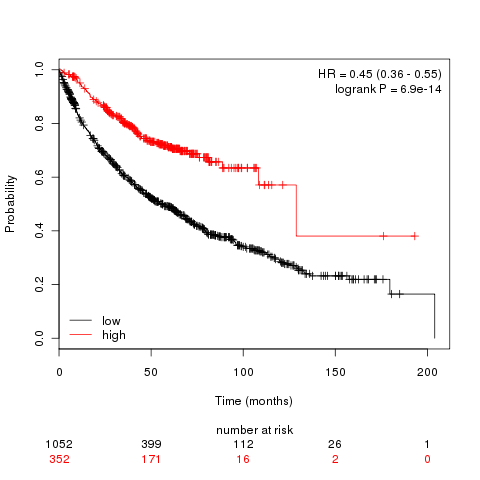

Supplement: Figure S2 — Kaplan-Meier plot of the unified lung cancer patient cohort available at the Kaplan-Meier Plotter web tool. x-axis indicates event-free survival time. y-axis represents the percentage of patients event-free survival. The black line represents patients with lower MAX expression, and the red line, patients with higher MAX expression. Crosses mark censored data. (TIFF) [file pone.0082457.s002.tiff]

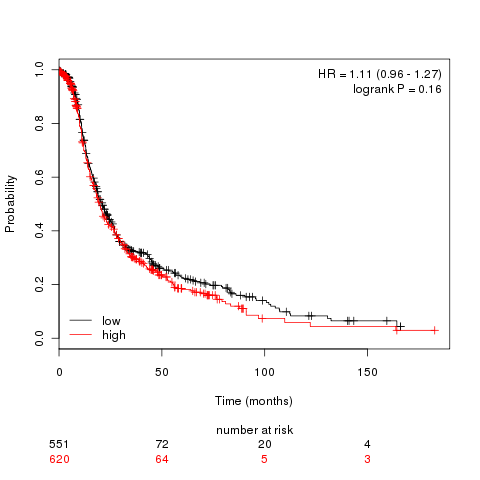

Supplement: Figure S3 — Kaplan-Meier plot of the 2013 ovarian cancer patient cohort available at the Kaplan-Meier Plotter web tool. x-axis indicates event-free survival time. y-axis represents the percentage of patients event-free survival. The black line represents patients with lower MAX expression, and the red line, patients with higher MAX expression. Crosses mark censored data. (TIFF) [file pone.0082457.s003.tiff]

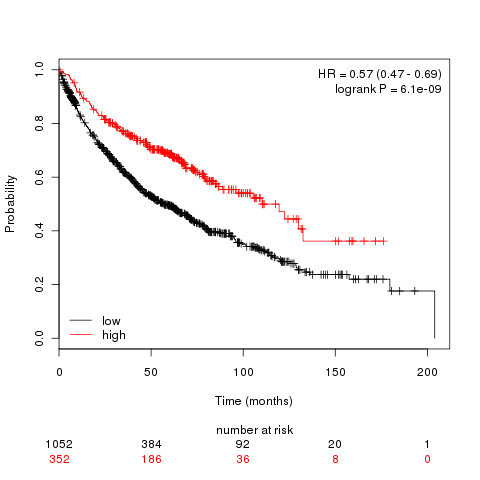

Supplement: Figure S4 — Kaplan-Meier plot of the 2012 breast cancer patient cohort available at the Kaplan-Meier Plotter web tool. x-axis indicates event-free survival time. y-axis represents the percentage of patients event-free survival. The black line represents patients with lower TFEC expression, and the red line, patients with higher TFEC expression. Crosses mark censored data. (TIFF) [file pone.0082457.s004.tiff]

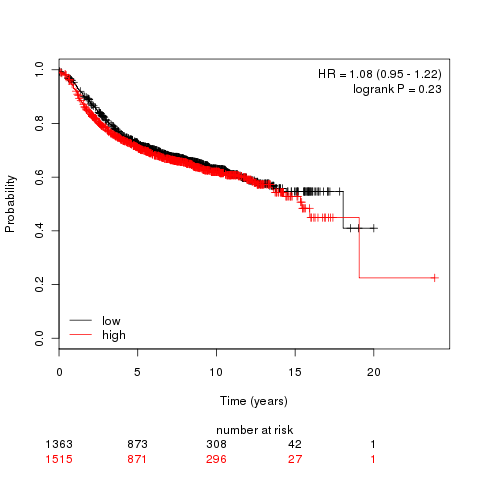

Supplement: Figure S5 — Kaplan-Meier plot of the lung cancer patient cohort available at the Kaplan-Meier Plotter web tool. x-axis indicates event-free survival time. y-axis represents the percentage of patients event-free survival. The black line represents patients with lower TFEC expression, and the red line, patients with higher TFEC expression. Crosses mark censored data. (TIFF) [file pone.0082457.s005.tiff]

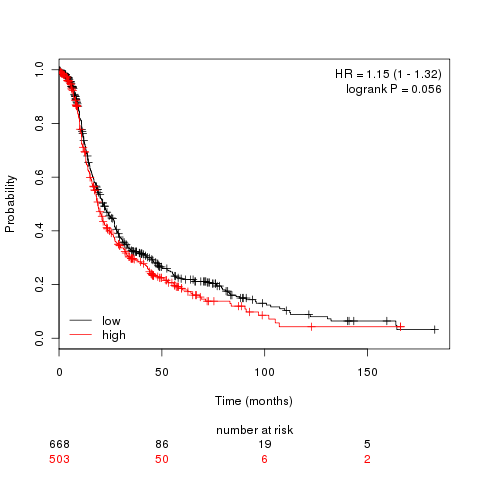

Supplement: Figure S6 — Kaplan-Meier plot of the 2013 ovarian cancer patient cohort available at the Kaplan-Meier Plotter web tool. x-axis indicates event-free survival time. y-axis represents the percentage of patients event-free survival. The black line represents patients with lower TFEC expression, and the red line, patients with higher TFEC expression. Crosses mark censored data. (TIFF) [file pone.0082457.s006.tiff]
